# Active Polylactic Acid (PLA) Films Incorporating Almond Peel Extracts for Food Preservation

**DOI:** 10.3390/molecules30091988

**Published:** 2025-04-29

**Authors:** Laia Martin-Perez, Carolina Contreras, Amparo Chiralt, Chelo Gonzalez-Martinez

**Affiliations:** Institute of Food Engineering Food UPV, Universitat Politecnica de Valencia, Camino de Vera s/b, 46022 Valencia, Spain; lmarper1@upv.es (L.M.-P.); caconmo1@upvnet.upv.es (C.C.); dchiralt@tal.upv.es (A.C.)

**Keywords:** orange juice, sunflower oil, mechanical and barrier properties, HFM, phenols

## Abstract

Almond peel extracts, containing 0.2–0.8% (*w*/*w*) phenolic compounds with notable antioxidant and antimicrobial activities, could be used as a natural source of active compounds for the development of active films for food preservation. In this study, almond peel extracts obtained by subcritical water extraction at 160 and 180 °C were incorporated into PLA films (PLA-E160 and PLA-E180). The films were characterized in terms of their microstructure, mechanical, barrier, optical and thermal properties. Furthermore, the release of phenolic compounds and hydroximethylfurfural (HFM) into food simulants with different polarity was evaluated, as well as the film’s potential antioxidant and antimicrobial activities. To validate their effectiveness as active packaging materials, shelf-life studies were conducted on fresh orange juice and sunflower oil packaged using PLA-160 films. The results show that the incorporation of the almond peel extracts led to significant changes in the films’ microstructure and mechanical properties, which became darker, mechanically less resistant, and stretchable (*p* < 0.05), with slightly lower thermal stability than neat PLA films. The release of phenolic compounds and HFM from extract-enriched films was promoted in the 95% ethanol simulant due to the matrix swelling and relaxation. Food products packaged with PLA-E160 exhibited slower oxidative degradation during storage, as indicated by the higher ascorbic acid content and hue color in orange juice and lower peroxide content in sunflower oil. Nevertheless, both in vivo and in vitro studies showed no antimicrobial effectiveness from the films, likely due to the limited release of active compounds to the surrounding medium. Thus, almond peel extract conferred valuable properties to PLA films, effectively reducing oxidative reactions in food products sensitive to these deterioration processes.

## 1. Introduction

Agricultural and food industry wastes, such as residues from fruits, vegetables, and grain processing, as well as animal husbandry, pose significant environmental challenges. Poor management of agri-food waste can lead to severe environmental issues, such as water pollution, soil degradation, and greenhouse gas emissions. Furthermore, mismanaged waste represents a loss of valuable biomass that could be utilized for energy production, composting, or material recovery. To mitigate these issues, stricter regulations are being implemented to promote the sustainable management of agri-food wastes, encouraging recycling and valorization practices. The almond industry generates substantial lignocellulosic residues, including almond skin or peel, hulls, and shells. Almond peel, which accounts for 6–8% wt of the seed, is well-known for its rich content of bioactive compounds, mainly flavonoids (i.e., catequin), phenolic acids (mainly hydroxybenzoic, hydroxycinnamic and caffeic acids), and proanthocyanidins [1]; these compounds exhibit different antioxidant and antimicrobial activities [2,3,4,5]. Considering their composition and rich bioactive profile, these residues represent a valuable resource, which has been explored for various applications, such as the development of dietary supplements, food preservatives, cosmetics, and the development of active packaging materials.

Active packaging materials incorporating polyphenol-rich extracts are gaining increasing attention, as they could contribute to inhibiting microbial growth and the oxidative degradation of foods. Several studies have demonstrated the efficiency of using phenol-loaded films in extending the shelf life of different foods such as meat products, oils, and fish, among others [6,7]. Furthermore, the use of biodegradable polymers to develop active packaging materials provides an eco-friendly alternative to conventional petroleum-based plastics. Among these biodegradable polymers, polylactic acid (PLA), derived from the fermentation of corn starch and other polysaccharide sources, is one of the most commercially available biomaterials. The implementation of such innovative packaging solutions could significantly reduce food waste while contributing to more sustainable food systems and to plastic-free environments.

The valorization of lignocellulosic residues typically involves the fractionation of valuable components through various extraction processes. Conventional extraction methods, such as solvent extraction, are commonly used but have limitations in terms of sustainability and efficiency. Emerging green extraction techniques, such as subcritical water extraction (SWE), offer more environmentally friendly and efficient alternatives. SWE utilizes liquid water at high temperatures and pressures below its critical point (374.15 °C and 22.1 MPa) as a solvent, being highly effective for extracting medium- and low-polarity components such as polyphenols [8]. Several studies have reported the successful application of SWE to extract phenolic compounds from various agri-food wastes, such as onion skin and kiwifruit peel, with the resulting extracts exhibiting remarkable antioxidant and antimicrobial activities [9,10,11]. Some authors obtained phenolic-rich extracts from almond peel by using SWE at 160 and 180 °C, with the latter showing the greatest antioxidant potential and antibacterial capacity against *L. innocua* and *E. coli* [8].

At high extraction temperatures, new compounds could also be formed derived from sugar caramelization and Maillard reactions, such as 5-hydroximethyl furfural (HMF) [12,13,14], especially when processing agri-food residues with a high sugar content. HMF is almost ubiquitous in commonly consumed thermally processed foods, such as breakfast cereals, bread, dairy products, and fruit juices. Many studies have demonstrated its negative effects on human health, including cytotoxicity on the mucous membranes, skin, and upper respiratory tract, mutagenicity, chromosomal aberrations, and carcinogenicity in both humans and animals [15]. However, more recent studies have reported that HMF exhibits a wide range of positive effects, such as antioxidant, anti-allergic, anti-inflammatory, anti-hypoxic, anti-falciform, and anti-hyperuricemic effects [15]. These beneficial or harmful effects largely depend on its concentration. Humans are estimated to consume between 30 and 150 mg of HMF per day through various food sources, but safe intake levels have not been clearly defined. The Codex Alimentarius Standard has established an upper limit for HFM to be considered safe for consumption. Therefore, beyond the benefits that active compounds provide to packaging materials, the presence of other compounds that may impact the safety of the packaged food must be carefully evaluated.

This study aims to evaluate the effect of incorporating phenolic-rich extracts from almond peel, obtained by subcritical water extraction, on the functional properties of PLA films in terms of microstructure, mechanical, barrier, optical, and thermal properties. It also assesses the release of phenolic compounds and HFM from the packaging material into different food simulants, as well as its effectiveness in limiting oxidative reactions and microbial spoilage in various food products, such as sunflower oil and fresh orange juice.

## 2. Results and Discussion

### 2.1. Film Microstructure

**Figure 1:**
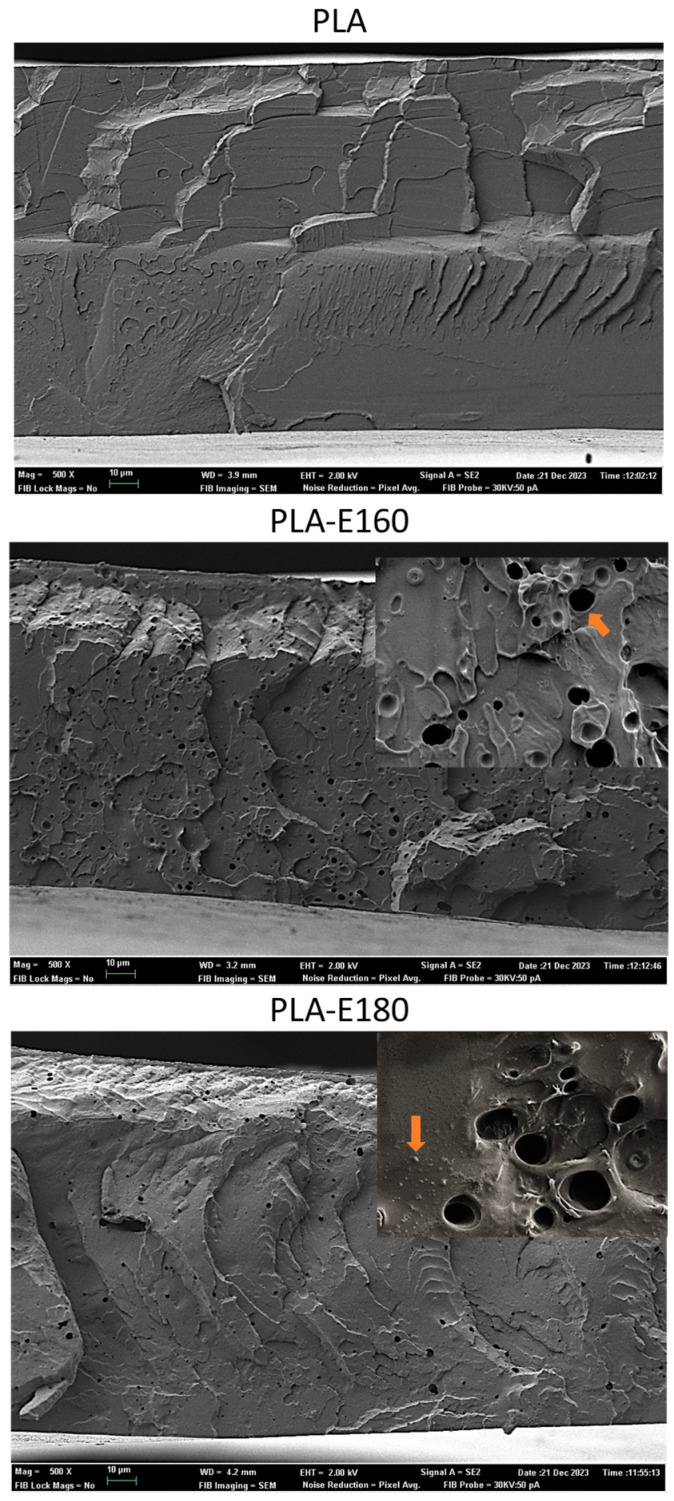
Cross-section FESEM micrographs of pure PLA and PLA films incorporating E-160 and E-180 extracts. Arrows: aggregates, cavities.

Figure 1 shows the cross-section of the different PLA films obtained by FESEM. Neat PLA films exhibited the typical morphology of amorphous polymers, showing brittle and rubbery domains [6]. The microstructure of PLA films remarkably changed after the extracts’ incorporation, becoming more heterogeneous and exhibiting more brittle sections, especially the PLA-E180 films. In all cases, small dispersed particles and concavities in the PLA matrix were observed, which are more evident in the magnified micrographs. The presence of these small aggregates suggests a lack of total miscibility of the extract compounds with the PLA matrix. Table 1 shows the main components of the extracts, including phenols, proteins, ashes, and carbohydrates, mainly constituted by free sugars, and partially hydrolyzed hemicelluloses, whose extraction was favored by SWE [16]. Most of these compounds are not miscible with molten PLA and remain dispersed in the polymer matrix. Other authors also observed particle aggregates in PLA films blended with certain phenolic acids [17] or rice straw extracts [6].

Additionally, small holes (around 1 μm diameter) were also detected in the polymer matrix, pointing to the formation of volatiles (CO_2_) during the films thermoforming. This may be associated with the thermal degradation of the sugars present in the extracts during the film processing. In fact, TGA of previously reported extracts [8] revealed a mass loss step (about 10% mass loss) within the 40–180 °C range, corresponding to the bound water loss of the extracts and the caramelization of free sugars, which implies volatile generation [8]. It is likely that this sugar degradation also occurred during the thermocompression step completed at high temperature (160 °C) and pressure (100 bars) to obtain the films, promoting the release of volatiles within the PLA matrix. These holes were also observed by Freitas et al. [6] in thermoprocessed PLA films with rice straw extracts.

The microstructural changes induced by the extracts’ incorporation into the PLA films could affect their functional properties. Both miscible and dispersed extract components may affect the cohesive forces of the polymer matrix, with great influence on mechanical and barrier properties. Likewise, diffusion processes from the films, which are mainly responsible for their active properties, would be only expected for homogeneously blended molecules with PLA.

### 2.2. Physical Properties of the Films

#### 2.2.1. Film Optical Properties

**Figure 2:**
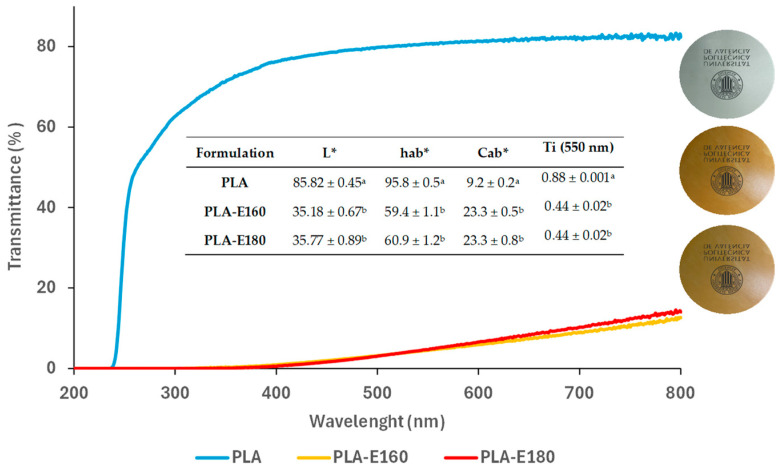
Visual appearance, luminosity (L*), hue (h_ab_*), chrome (C_ab_*), internal transmittance at 550 nm (Ti), and UV-vis spectra of the neat PLA and PLA films incorporating E-160 and E-180 extracts. ^a,b^ Different letters in the same column indicate significant differences between films (α = 0.05).

The optical properties of the films are shown in Figure 2 in terms of color coordinates (L*, h_ab_* and C_ab_*) and internal transmittance at 550 nm as a measure of the film’s transparency, together with the UV-vis transmittance spectra and visual appearance. Pure PLA films were colorless and highly transparent, whereas extract-enriched films were less transparent (lower Ti values), with a brownish, darker, and more saturated color (lower hue angle and lightness and greater chrome values, respectively) compared to neat PLA (*p* < 0.05), regardless of the extract incorporated. This effect is attributed not only to the intrinsic color of the initial extracts but also to the neoformation of colored compounds, such as 5-hidroxymetylfurfural (HFM), during the thermal treatments. These reactions occur because of the Maillard and caramelization reactions promoted under high-temperature and -pressure conditions (i.e., subcritical water extraction and thermo-compression processes). This hypothesis was confirmed by the quantification of the HFM content in the initial extracts and the HMF in the films released into different simulants, as discussed in the following sections. Furthermore, the extract-rich films exhibited a strong UV-light absorption capacity in the 200–400 nm wavelength range, blocking around ~93% of UV light compared to the control. This is attributed to the strong UV-absorbing properties of the aromatic structures and functional groups present in the flavonoids and polyphenolic compounds in the extracts, making them natural broad-spectrum blockers [18].

#### 2.2.2. Mechanical and Barrier Properties

Table 2 shows the mechanical parameters (elastic modulus, tensile straight, and percentage of elongation at break), together with the water vapor and oxygen permeabilities of the films. The mechanical and permeability values of the neat PLA films agree with those reported by other authors [6,19,20]. PLA-E160 and PLA-E180 films were significantly (*p* < 0.05) less rigid, resistant to breaking, and stretchable (lower EM, TS, and E% values) than neat PLA (control film) due to the presence of the previously observed structural discontinuities, leading to weaker films. This effect could be explained by the following: (1) the reduction in PLA–PLA intermolecular forces caused by the intercalation of the extracts’ components between the polymer chains; (2) the presence of discontinuities (holes and aggregates) throughout the continuous polymeric matrix; and (3) by the partial hydrolysis of the PLA chains during the thermocompression step. Similar trends have been already observed by other authors, to a lesser or greater extent, when incorporating different active compounds into PLA films, such as plant extracts (rice straw, Allium spp., waste orange peel, rosemary ethanolic), phenolic acids, and oregano essential oil, among others [6,19,20,21]. These changes were minimal in PLA-E160 films (reduction of 14.5% and 9% in mechanical resistance and extensibility in comparison with the control film). However, they were more noticeable when incorporating the extract obtained at the higher temperature (E-180), showing a decrease of about 83% and 84% in the TS and E% values. This indicates that the extraction temperature significantly affects the mechanical properties of the films due to differences in their composition, especially in terms of phenolic content (Table 1) and neo-formed compounds (commented on below), both of which were higher in the PLA-E180 films.

In contrast, the water content, thickness, and barrier properties of the films were not compromised due to the extract’s addition. In general, no significant differences (*p* > 0.05) were found in the water vapor and oxygen permeability values between neat PLA and PLA films incorporating the extracts, except for the PLA-E180 films, which showed slightly lower OP values (*p* < 0.05). This reduction could be attributed to its greater antioxidant activity, which may act as an oxygen scavenger, thereby decreasing oxygen permeation through the film. This effect has been previously reported by other authors when incorporating antioxidant compounds into different film matrices [20,22,23].

#### 2.2.3. Thermal Behavior: Thermal Stability and Tg Values

**Figure 3:**
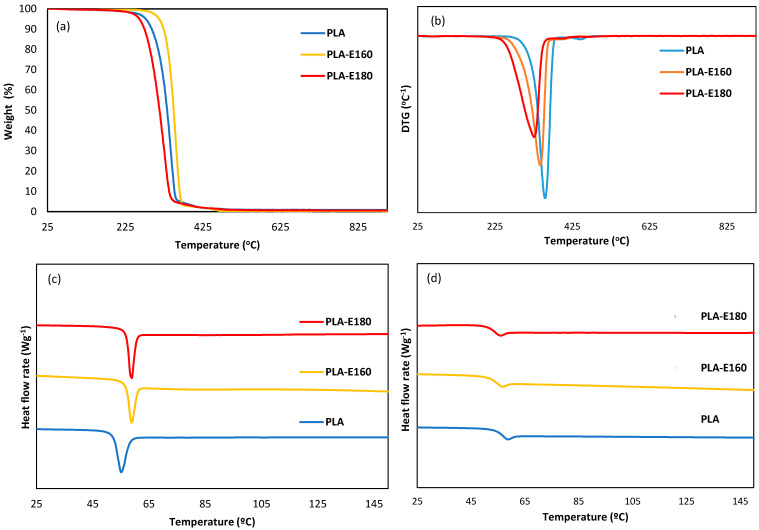
TGA (**a**) and DTG derivative curves (**b**), and DSC thermograms obtained from the 1st (**c**) and second (**d**) heating of the neat PLA films and those incorporating E-160 and E-180 extracts.

Figure 3 shows the TGA from 25 to 600 °C, derivative curves, and DSC thermograms of the different PLA-based films during the first and the second heating scans. Table 2 summarizes the thermal degradation temperatures of the films. TGA revealed differences in the thermo-degradation profile of the polymer due to the incorporation of the extracts. The onset degradation temperature (T_onset_) of amorphous PLA was around 323 °C, with a single step of weight loss (maximum at 354 °C), associated with the polymer matrix decomposition [24]. These degradation temperatures significantly decreased (*p* < 0.05) due to the extract incorporation, in agreement with the presence of low-molecular-weight compounds (phenols, sugar, sugar, and minerals) with lower thermal stability [8] (Freitas et al., 2023); this effect was more pronounced in the extract obtained at the highest temperature.

The glass transition temperature (Tg) of neat amorphous PLA was within the range previously reported by other authors [24,25]. The incorporation of the extract did not significantly affect the Tg values of PLA films (*p* > 0.05), although a trend towards lower Tg values was observed, likely due to partial PLA hydrolysis during the film processing. The Tg values in the second heating step (Tg_2_) were lower due to the water loss during the first heating.

### 2.3. Bioactive Potential of the Films

The bioactive potential of the active films depends on the concentration of the active compounds, their antimicrobial and/or antioxidant powder, and their ability to be released from the polymeric film into the surrounding medium. In this study, all these issues were considered through in vitro and in vivo analyses. The in vitro studies assessed the antioxidant activity of the films by evaluating the release of both phenols and hydroxymethylfurfural (HFM) into food simulants with different polarity and pH levels, as well as their potential antimicrobial capacity. HMF was formed in the extracts during the SWE process due to the high temperatures applied and the presence of free sugars and peptides. Other authors reported HMF formation when using SWE on different food by-products, such as artichoke leaves, lemon peel, or flaxseed meal [14]. HMF has been reported to exhibit antioxidant and antibacterial properties but also potential toxicity [15,26], hence the importance of controlling its release in food products. Likewise, phenolic compounds are well-known for their antioxidant and antimicrobial capacities [27,28]. To validate the effectiveness of the developed packaging materials, in vivo studies were conducted by assessing different quality parameters of fresh orange juice and sunflower oil packaged with these films.

**Figure 4:**
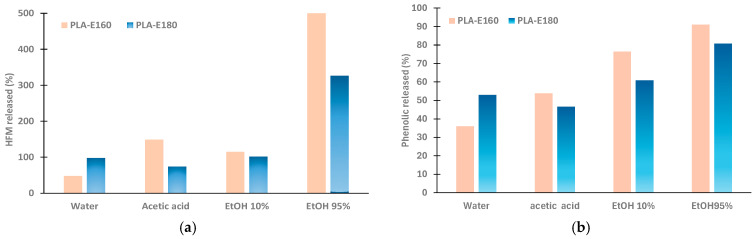
Percentage of HMF (**a**) and phenolic compounds (**b**) released from PLA films incorporating E-160 and E-180 almond peel extracts to the different simulants.

The HFM concentrations in the initial extracts were 0.168 ± 0.001 and 0.394 ± 0.014 (g/100 g extract) for E-160 and E-180, respectively. These values are notably comparable to those reported in other thermally processed food products, such as coffee and pomegranate juice [29]. Nevertheless, an increase in this HFM content was expected during the film processing, as intense heat treatment can enhance its formation. Figure 4 shows the percentages of HMF in the films released into the different food simulants, which was calculated from the theoretical HMF content in the film, determined as described in Section 3.4. These percentages markedly depended on the type of simulant used. The polymer–simulant interactions, which cause the respective swelling and relaxation of the polymer matrix, largely determine the amount compound released. In contact with water, the relaxation of the hydrophobic PLA matrix was limited, and therefore also the diffusion of the non-polymeric compounds into the liquid medium. However, as the polarity of the simulant increased (i.e., 95% ethanol), the simulant easily penetrated the film, leading to swelling and relaxation of the matrix, thus promoting the diffusion and release of the trapped molecules. Some authors have even reported the partial degradation of PLA chains upon contact with ethanolic solutions, thus further enhancing the release of compounds [30,31]. In fact, a greater HMF release ratio was reached in 95% ethanol, where the compound diffusion process was favored.

It is remarkable that in most of the cases, the percentage of HMF exceeded 100% (Figure 4), which indicates that the theoretical HMF content in the films estimated on the basis of that incorporated in the extract underestimates the actual HMF in the films and confirms the additional HMF formation during thermoprocessing. Nevertheless, the total migration of HMF from the films in each simulant (Table 3) was relatively low, compared to that found in many commercial foods [32,33]. In Table 3, the HFM released was expressed as mg/kg foodstuff (ppm), considering a 1 dm^3^ cubic package, for comparison purposes with the usual concentration units in foods. Migrated levels are important from the point of view of safety issues in food contact packaging materials. The highest values were observed in the 95% ethanol simulant of PLA-E160 and PLA-E180 films (0.35 ± 0.04 and 0.474 ± 0.002, respectively), which were below the levels found in many commercial foods [32,33]. No HMF release was detected when using the isooctane as a simulant.

Therefore, from the point of view of the potential migration of HMF to food, it is concluded that PLA films with almond peel extracts obtained in subcritical water at 160 °C and 180 °C can be considered safe. A more noticeable HMF migration could be expected in high-fat foods (simulated by 95% ethanol), with these values remaining very low compared to those found in commonly processed foods consumed daily.

Likewise, Figure 4b also shows the percentage of phenolic release into the different simulants, which was also affected by the type of simulant. These values ranged between 40 and 50% (for water and acetic acid) and 80–90% (for ethanolic-based simulants), and the effect of the type of simulant followed the same trends as that observed for the migrated HFM. These results highlight the potential of these films to be used as antioxidant packaging materials for food products susceptible to oxidation. Nevertheless, it could also be interesting to further validate their effectiveness in high-moisture, low-pH foods, such as orange juice.

Regarding the in vitro antimicrobial study of the films, the growth of different bacteria (*L. innocua*, *E. coli* and *S. aureus*) in the respective medium in contact with the extract-rich PLA films was similar to that found in the control samples. Thus, all the samples showed growth over 7 logs (CFU/mL), with the extract-loaded films exhibiting a mild antimicrobial effect likely due to the low amount of extract released to the aqueous culture medium. Similar results were obtained by other authors working with non-defatted almond peel and *L. monocytogenes* and *Salmonella enteritidis* [34]. On the contrary, these authors observed a defining inhibitory activity against *S. aureus*, which was attributed to the presence of fatty acids from the almond peel such as linoleic, palmitoleic, and oleic acids with antimicrobial potential. Despite the low antimicrobial activity observed in the PLA films incorporating the defatted almond peel extracts, it is crucial to validate the results in real food products, where the initial microbial contamination is usually much lower than that used in the in vitro antimicrobial tests (10^6^ CFU/mL).

Concerning the in vivo studies, the effectiveness of the active packaging material in preserving oxidation and microbial growth in real food systems, such as fresh orange juice and sunflower, oil was analyzed. To this aim, only mono-dose bags of PLA containing 6% of the E160 extract (PLA-E160) were used due to the high brittleness of the PLA-E180 films, which severely affected their heat-sealing capacity.

### 2.4. Film’s Ability to Preserve Fresh Orange Juice

**Figure 5:**
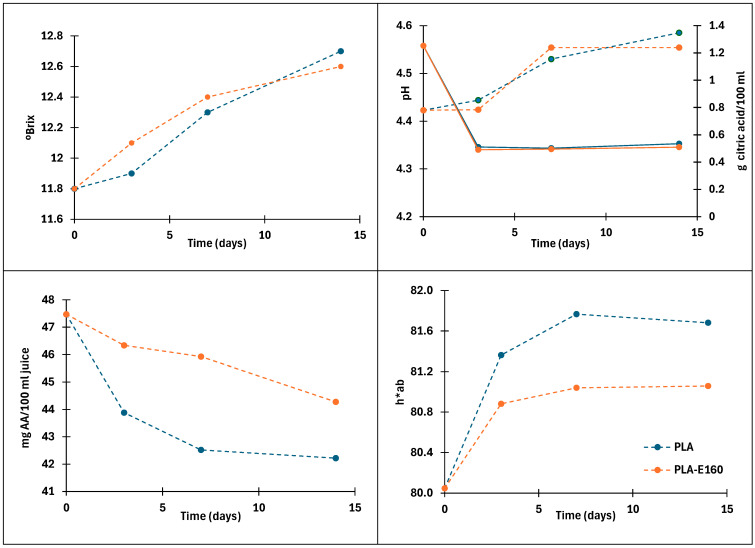
Brix, pH (solid lines), total acidity (dashed lines), ascorbic acid (AA) content, and hue color values of fresh orange juices packaged with PLA and PLA-E160 films through cold storage.

The ascorbic acid (AA) content of juice packed in PLA and PLA-E160 films throughout the storage period is shown in Figure 5, together with the pH, total acidity, and color changes quantified by the hue values. The fresh orange juice initially presented a high content of l-ascorbic acid, 47.5 mg AA/100 mL juice, a value in the range reported by other authors [35]. This content can vary depending on the processing conditions, time, and type of storage, as AA is degraded by oxygen, pH, enzymes, light, the presence of metal catalysts, and high temperatures [36,37]. As can be seen, gradual loss of ascorbic acid occurred over time in both samples. These results are consistent with those obtained in orange juice packaged in low-density polyethylene bags containing (or absent of) different amounts of green tea extract [35]. According to several authors, this decrease is mainly attributed to the presence of dissolved oxygen in the juice and in the headspace of the packaging at the beginning of storage [38,39,40,41]. Throughout storage, oxygen permeation through the container significantly contributes to the advancement of the aerobic oxidation process, resulting in a high rate of ascorbic acid oxidation, leading to its reduction during storage [42]. The degradation of AA during cold storage mainly results in carbonyl compounds such as aldehydes, ketones, and carboxylic acids [38]. The formation of these compounds is of particular importance, as they can influence some juice properties including color, pH, and acidity.

The sample packed with almond peel extract showed the highest AA values throughout the entire storage period (*p* < 0.05). The degradation kinetics of the remaining AA concentration in the juice followed a practically linear trend (r^2^ = 0.89) during the first seven days, with degradation rate values of 0.7 and 0.2 (mg AA/100 mL and day) for the juice packed in PLA and PLA-E160, respectively. The slower degradation rate of AA in the active films can be attributed to the strong light barrier capacity of the PLA-E160 film, which blocked around 93% of UV-transmitted light, as well as to the release of antioxidant compounds that limit the oxidative degradative processes of sensitive compounds. This latter contribution could also be remarkable, as around 50% of the phenolic compounds are expected to be released, based on the in vitro results obtained for the films immersed in the low-pH simulant. In contrast, the effect of the oxygen barrier capacity of the active films is negligible, as no significant differences were found between the OP values of the control and PLA-E160 films (Table 2).

The freshly squeezed juice presented an initial total acidity value of 1.2 g citric acid/100 mL, pH = 4.42 and 11.8 °Brix, in agreement to that found by other authors [43]. Organic acids play a crucial role in the distinctive taste and palatability of orange juice as a result of biochemical processes or fermentations produced by the development of microorganisms during storage, with citric acid being the most abundant, followed by malic acid [44]. Acidity values decreased gradually over time until the third day of storage (*p* < 0.05) in both samples, remaining relatively constant thereafter, while the pH slightly increased up to values of 4.6 (*p* < 0.05). Some authors attributed the decrease in total acidity values to the degradation of ascorbic acid [45]. On the contrary, other authors reported an increase in the total acidity and Brix values of the juice throughout storage, mainly associated with a remarkable growth of molds and yeasts that used easily fermentable free sugars for their growth, generating organic acids at the same time during juice storage [35]. These conditions were not observed in the juice analyzed in this study, as no molds or yeasts, which have a very high hydrolytic and fermentative potential, were detected. On the other hand, soluble solids increased progressively (*p* < 0.05) up to values of 12.6 °Brix throughout the storage time, regardless of the type of packaging used (*p* > 0.05).

The color of orange juice is mainly due to the presence of pigments such as carotenoids, while color changes are mainly associated with the degradation of ascorbic acid and carotenoids. Ascorbic acid degradation results in brown pigments [46,47]. Similarly, carotenoids are also susceptible to oxidation and isomerization reactions due to their unsaturated structure, leading to the formation of colorless compounds [48]. This degradation of carotenoid compounds, especially violaxanthin, anteroxanthin, and the isomerisation of 5.6 epoxy-carotenoids to 5.8 epoxy, leads to an increase in hue values [35,46]. Additionally, the presence of endogenous enzymes such as pectinmethylesterases in non-heat-treated juices contributes to changes in turbidity, which could affect the color of the samples [46]. The development of the hue values throughout storage is shown in Figure 5, whereas the values of the chromatic parameters of lightness (L*), hue (h*_ab_) and chroma (C*_ab_) are shown Table S1 (Supplementary Materials), along with the color difference with respect to the fresh juice at t = 0. Both the control and active packaged samples showed a decrease in lightness and an increase in the hue values throughout storage, resulting in darker samples with a yellowish-brown appearance. No significant differences were found in the lightness and chroma values between samples at the end of storage (*p* > 0.05). However, the increment in the hue values was significantly lower (*p* < 0.05) in samples packaged with the active film (Figure 5), likely due to the antioxidant activity of the almond extract, which limited carotenoids and ascorbic acid degradation. The color differences (ΔE) increased progressively over time (*p* < 0.05), with the values of the samples packaged with the active films being slightly lower than those in the control.

Regarding the microbial analysis of the refrigerated samples, no mold or yeast growths were detected throughout the storage time. The total bacteria counts slightly increased from 7 to 8.2 and 9 UFC/mL after 14 days of cold storage in orange juice packaged in the control and PLA-E160 films, respectively. Thus, no antimicrobial effect was observed in samples packaged with the active films. 

### 2.5. Film’s Ability to Prevent Sunflower Oil Oxidation

**Figure 6:**
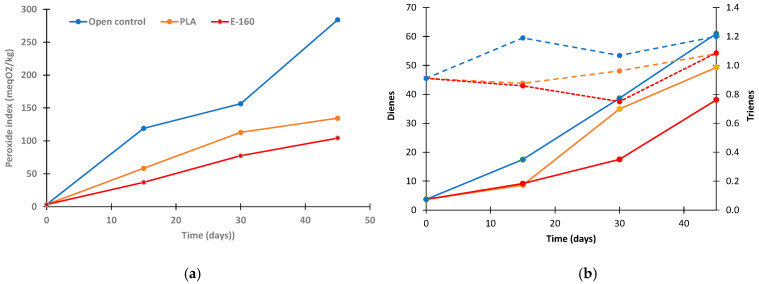
Peroxide index (meq O_2_/kg) (**a**), conjugated dienes (full lines), and conjugated trienes (dash lines) (**b**) (in g/100 g) of sunflower oil packaged with PLA, PLA-E160 films, and in a glass Petri dish (open control).

The protective power of PLA-160 packaging on sunflower oil was evaluated by means of the peroxides index (PI), dienes, and trienes throughout 45 days at 30 °C and 53% RH (Figure 6), under UV light exposure to promote oxidative processes. As controls, neat PLA bags and oil placed in an uncovered glass Petri dish were used. PI values are associated with the presence of peroxides derived from polyunsaturated fatty acids, thus indicating the initial oxidation level of the sample, while hydroperoxides are formed as primary oxidation products, which can be further degraded into secondary oxidation products [7].

As can be observed, the initial PI value of sunflower oil was 3.03 mEq O_2_/kg, which is consistent with the values found in other studies [7]. PI values increased significantly (*p* < 0.05) in all cases, reaching values of 156, 112, and 77 meq O_2_/kg after 30 days of storage for open control, the PLA-packed oil, and the oil packed in PLA-E160, respectively. These values were significantly higher (*p* < 0.05) in the open control due to the oxidative conditions of the test, while in the PLA-packed oil, peroxide formation was slower as the packaging material limited oxygen transfer. The PLA-160 samples exhibited the lowest PI values at both 30 and 45 days of storage (*p* < 0.05), in agreement with the antioxidant potential of the almond peel extract.

Conjugated dienes and trienes are formed by the rearrangement of hydroperoxide double bonds during oxidation. Dienes represent the primary degradation products of the oil and could be used to confirm the peroxide content, while conjugated trienes are related to the secondary products of oxidation [49,50]. The initial contents of conjugated dienes and trienes were 3.7 g/100 mL and 0.91 g/100 mL, respectively. All samples experienced an increment in conjugated dienes proportionally to the rise in PI values. However, these values were 23% lower in the PLA-E160 samples after 45 days of storage (*p* < 0.05), indicating a reduction in the occurrence of primary degradation products. In contrast, conjugated trienes remained constant throughout the storage period in all samples, regardless of the packaging used, reaching an average value of 0.94 ± 0.22 g/100 mL. These results suggest that active packaging material limits the formation of primary degradation products in sunflower oil, while the second stage of degradation is inhibited [51]. Therefore, incorporating active extracts from the almond peel agri-food residue into PLA mono-dose bags provides effective protection against oil oxidation during storage.

## 3. Materials and Methods

### 3.1. Materials

Almond peel (AS) (*Prunus dulcis*, *Nonpareil* var., CA, USA) was kindly provided by Importaco S.A. (Valencia, Spain) from their 2022 harvest. Aqueous extracts (E) were sub-critically water-extracted using 160 and 180 °C and 7 and 15 bars, respectively, according to the method described by [8]. The aqueous extracts were then lyophilized (E-160, E-180) and stored in desiccators (P_2_O_5_, 0% relative humidity (RH)) at 4 °C until further use. The characterization of these extracts, in terms of the total phenol content (TPC), antioxidant capacity (expressed as the efficient concentration, EC_50_), and minimum inhibitory concentration (MIC) of the extracts is summarized in Table 1. Navel oranges (var. Late) and sunflower oil were purchased from a local supermarket.

Amorphous PLA 4060D, with a molecular weight of 106,226 and a density of 1.24 g/cm^3^, was obtained from NatureWorks (Plymouth, MN, USA). Magnesium nitrate (Mg(NO_3_)_2_), sulfuric acid (H_2_SO_4_ 2 M, 98%), ascorbic acid (99%), sodium hydroxide (NaOH 0.1 N), chloramine-T (0.005 M), sodium thiosulfate pentahydrate (Na_2_S_2_O_3_·5H_2_O), isooctane (C_8_H_18_, 99%), and phosphorus pentoxide (P_2_O_5_) were from PanReac Química S.L.U. (Castellar del Vallès, Barcelona, Spain), while potassium iodide (KI) was acquired from Acros Organics^®^ (Geel, Belgium). For microbiological assays, peptone water (WP), Tryptic Soy Broth (TSB), plate count agar (PCA), and bacteriological agar were purchased from Scharlab (Barcelona, Spain). The *Staphylococcus aureus* strain (CECT 8148) was supplied by the Spanish Type Culture Collection (CECT, Universitat de València, València, Spain).

### 3.2. Film Preparation

PLA films with (PLA-E160 and PLA-E180) and without extract (PLA, control) were prepared by melt blending and compression molding, incorporating 6% of the extracts into the mixture. The amorphous PLA pellets were pre-ground using a grinder (IKA, model M20, Staufen, Germany) and preconditioned in P_2_O_5_ for 2 days until constant weight was achieved to remove the residual water. The ground PLA and extracts were melt-blended using an internal mixer at 160 °C and 50 rpm for 7 min (HAAKETM PolyLabTM QC, Thermo Fisher Scientific, Karlsruhe, Germany). The blends were cryo-milled using liquid N_2_ (IKA, model M20, Staufen, Germany) and stored in desiccators with P_2_O_5_. Once conditioned, 4 g of each formulation was thermo-processed using a hot-plate hydraulic press (Model LP20, Labtech Engineering, Samut Prakan, Thailand) to obtain the films, by applying 1 min of preheating at 160 °C, 3 min of heating at 100 bar and 160 °C, and 5 min of cooling (without pressure) until 70 °C. The resulting films were stored in a desiccator with a saturated Mg(NO_3_)_2_ solution (53% RH).

### 3.3. Film Characterization

#### 3.3.1. Microstructure

In order to observe the morphology of the cross-section of the films, a field emission scanning electron microscope (FESEM, Ultra 55, Zeiss, Oxford Instruments, Oxford, UK) with an acceleration voltage of 2 kV was used. To obtain the micrographs, the samples were cryo-fractured by immersion in liquid nitrogen and subsequently coated with platinum prior to observation.

#### 3.3.2. Moisture Content

The moisture content of the films was determined using a gravimetric method in duplicate. Film samples, conditioned at 25 °C and 53% RH for one week, were cut and subsequently dried at 60 °C until constant weights were reached and then later on placed in a desiccator at 0% RH for 3 days to ensure complete dryness.

#### 3.3.3. Mechanical Barrier and Optical Properties

The thickness of the films was measured using a digital micrometer (Palmer, COMECTA model, Barcelona, Spain, with an accuracy of 0.001 mm) at 8 random positions on the sample.

In accordance with ASTM D882 [52], a universal testing machine (Stable Micro Systems, TA.XT plus, Surrey, UK) was used to determine the tensile properties of the films. The mechanical behavior was analyzed in terms of the elastic modulus (EM, in MPa), tensile strength (TS), and percentage elongation at break (%E). To this aim, 25 mm × 100 mm preconditioned film samples (53% RH) were stretched at a crosshead speed of 50 mm/min until breakage, using two grips initially separated by 5 mm. Measurements were carried out in twelve samples per formulation.

The water vapor permeability (WVP) of the films was determined in triplicate gravimetrically, following ASTM E96/E96M [53]. The samples were cut into 3.5 cm diameter samples, placed, and sealed in Payne permeability cups containing 5 mL of distilled water (100% RH). The cups were then placed in desiccators equipped with fan and containing a saturated solution of Mg(NO_3_)_2_ (53% RH) at 25 °C. The permeability cups were weighed throughout the test period using an analytical balance (±0.00001 g), and the mass-loss rate was used to calculate the WVP once a stationary state was reached. 

The oxygen permeability (OP) of the films was determined following a modified version of the ASTM D3985-05 Standard Method [54]. An Ox-Tran Model 1/50 (Mocon, Minneapolis, MN, USA) was used at 25 °C and 53% RH to obtain the oxygen transmission rate (OTR). Two replicates per formulation were measured using 50 cm^2^ film samples exposed to an oxygen flow. OP values were calculated by dividing the OTR by the partial oxygen pressure difference between both sides of the film and multiplying by the average film thickness.

The optical properties of the films were also evaluated by measuring the UV-vis spectra from 240 to 800 nm using a UV-visible spectrophotometer (Evolution 201, Thermo Scientific, Waltham, MA, USA) in light transmission mode. The internal transmittance (T_i_) and CIEL*a*b* color coordinates were determined for the different samples using a CM-3600d spectrocolorimeter (Minolta Co., Ltd., Tokyo, Japan) as described by [6] by applying the Kubelka–Munk theory of multiple scattering. CIEL*a*b* color coordinates (lightness (L*), a* (red-green), and b* (yellow-blue) were calculated from the infinite reflectance spectra, using D65 illumination and a 10° observer. From the CIEL*a*b* coordinates, the chroma (C_ab_*) and hue (h_ab_*) were calculated (Equations (1) and (2)).

(1) $$C_{ab}^{*}=\sqrt{{\left(a\right)}^{2}+{\left(b\right)}^{2}}$$

(2) $$h_{ab}^{*}=arctan\left(\frac{a*}{b*}\right)$$

#### 3.3.4. Thermal Properties

The thermal properties of the films were studied using a differential scanning calorimeter (DSC, 1 Stare System, Mettler-Toledo, Greifensee, Switzerland) and a thermogravimetric analyzer (TGA 1 Stare System, Mettler-Toledo, Greifensee, Switzerland). Prior to analysis, the samples were cut into small pieces and conditioned in desiccators with P_2_O_5_ to eliminate residual moisture. For the DSC analysis, samples (approximately 10 mg) were placed in aluminum crucibles, sealed, and submitted to the following steps: heating from room temperature to 200 °C at 10 °C/min, holding at 200 °C for 5 min, cooling at 10 °C/min to −10 °C, holding at −10 °C for 5 min, and reheating to 200 °C at 10 °C/min. An empty aluminum crucible was used as a reference.

TGA was performed in samples (approximately 4–5 mg) placed in alumina crucibles and heated from 25 °C to 900 °C at 10 °C/min under a nitrogen flow (10 mL/min). The derived thermogravimetric (DTG) curves obtained were analyzed using STARe Evaluation Software version V12.00a (Mettler-Toledo, Greifensee, Switzerland). Both DSC and TGA thermal analyses were performed in duplicate.

### 3.4. Release of 5-Hidroxymetylfurfural (HMF) and Phenolic Compounds in Different Simulants

The release of active compounds (HMF and phenols) was performed in different food simulants, following the UNE EN 1186-1 standard for the migration of substances from plastic materials in contact with food. The tests involved the total immersion of the film samples in each simulant, using a surface-to-volume ratio of 1 dm^2^: 100 mL for 10 days at 40 °C, as specified by the UNE EN 1186-1 standard, except for iso-octane where the standard establishes 2 days at 20 °C. The food simulants used to emulate aqueous foods were as follows: A: distilled water, B: acetic acid 3% (m/V), and C: ethanol 10% (*v*/*v*). Ethanol 95% (*v*/*v*) and iso-octane were used to simulate fatty foods. After this time, the films were removed from the liquid phase, using a 0.45 μm pore-size filter. The liquid fractions were analyzed as to the 5-hidroxymetylfurfural (HMF) and total phenolic (TPC) contents.

HMF quantification was performed in the solid extracts (2% aqueous solutions) and in the simulant liquid phases after the film contact time, using high-liquid performance liquid chromatography (HPLC-UV, Agilent Technologies, model 1120 Compact 1200 LC, Palo Alto, CA, USA) equipped with a ZORBAX Eclipse Plus C18 column (4.6 × 150 mm, 5 μm particle size, Agilent Technologies, Palo Alto, CA, USA) and an UV detector (modelo, Agilent Technologies 1200 Series, Palo Alto, CA, USA). The system was controlled using the OpenLAB Data Analysis software (Build 2.203.0.573) (Agilent Technologies, Palo Alto, CA, USA). The test conditions were as follows: column temperature 35 °C, UV detector at 285 nm, and an isocratic mobile phase consisting of deionized water–acetonitrile (80:20) at a flow rate of 1.0 mL/min. A calibration curve (R^2^ = 0.9994) was previously obtained using standard HMF solutions of known concentrations. The theoretical HMF content of the films was estimated by considering the HMF content of the extracts and their mass fraction in the films (6% wt.)

The total phenol content (TPC) was quantified by the Folin–Ciocalteu method, in triplicate, by mixing 0.5 mL of the liquid fraction with 6 mL of distilled water and 0.5 mL of Folin reagent (2 N). After one minute, 1.5 mL of Na_2_CO_3_ at 20% (*w*/*v*) was added, with the final volume adjusted to 10 mL with distilled water and kept in the dark at 20 °C for 2 h. The absorbance was measured at 747 nm using a UV-Vis spectrophotometer (model Evolution 201, Thermo Scientific, Waltham, MA, USA). The total phenolic content was determined using a standard curve (R^2^ = 0.9991) of gallic acid and expressed as mg gallic acid equivalent (GAE) 100 g^−1^ extract. The theoretical TPC values of the films were estimated by considering the TPC content of the extracts and their mass fraction in the films (6% wt.).

The percentage releases of HMF and phenols in each simulant were determined from the amount released in each simulant and the corresponding theoretical total amount in the sample film.

### 3.5. Antibacterial Activity of the Films

The following methodology was followed based on previous studies employing Tryptic Soy Agar (TSA) as the culture medium [19,22]. Petri dishes (55 mm diameter) containing 10 mL of TSA were inoculated with a 100 µL of a bacterial suspension of *E. coli*, *L. innocua,* and *S. aureus*, at an initial concentration of 10^6^ CFU/mL. The plates were subsequently covered with the films, which had been previously sterilized in a laminar flow cabinet (Bio II advance, Telstar, Spain), sealed with Parafilm, and incubated at 10 °C for 6 days. After the incubation period, the contents of each plate were homogenized in 100 mL of buffered peptone water using a paddle blender (Masticator, IUL Instruments, Barcelona, Spain) for 3 min. Serial dilutions of each sample were then prepared and plated on selective media: violet red bile agar (VRBA) for *E. coli*, Palcam agar base with Palcam selective supplement for *L. innocua*, and Baird Parker agar base with RPF supplement for *S. aureus*. The plates were incubated at 37 °C for 48 h, after which the number of colonies was quantified and expressed as CFU/mL. All experiments were performed in triplicate, using uncovered Petri dishes as controls.

### 3.6. Ability of Active Films to Extend Shelf Life of Food

In vitro tests were conducted using two food matrices with different characteristics: freshly squeezed orange juice (acidic medium) and sunflower oil (rich in unsaturated fats). For this purpose, single-dose bags (11 × 9 cm) were obtained by vacuum heat sealing with a vacuum packer (SAECO Vacio Press Elite, Barcelona, Spain), using PLA (control) and PLA-160 films. Prior to use, the single-dose bags were exposed to UV light for 15 min on each side in a laminar flow cabinet (Bio II Advance, Telstar, Terrassa, Spain). The PLA-180 formulation was not suitable for bag production due to its high fragility and tendency to break during the thermo-sealing process.

#### 3.6.1. Physicochemical and Microbiological Analysis of Freshly Squeezed Orange Juice

The single-dose bags were filled with 10 mL of juice, obtained using a manual juicer and filtered through a 1 mm mesh. The bags were aseptically sealed and stored under refrigerated conditions (5 °C) in darkness for 0, 3, 7, and 14 days.

The quality of the orange juice was analyzed throughout cold storage by evaluating different physico-chemical and microbiological parameters. The ascorbic acid (AA) content was evaluated in duplicate using an automatic titrator (Titrotherm 859, Metrohm, Switzerland). Briefly, 4 mL of packaged juice was mixed with 100 mg of KI, 50 mL of distilled water, and 2 mL of H_2_SO_4_ and then titrated with 0.005 M chloramine-T. The pH and titratable acidity (TA) were measured in triplicate with a pH meter and automatic titrator (Titrotherm 859, Metrohm, Switzerland) using NaOH (0.1 N), respectively. The soluble solids content was determined at 25 °C using a refractometer (Hanna, HI 96801, Judetul Cluj, Romania). The color determination of the juice samples was performed in duplicate using a spectrocolorimeter CM-3600d (Minolta Co., Ltd., Tokyo, Japan), with a D65 illuminant and a 10° observer. The L* (lightness index), hue angle (h_ab_*) and chroma (C_ab_*) values were obtained, and the total color difference ΔE with respect to the freshly packaged sample was calculated using Equation (3):

(3) $$∆E=\sqrt{{\left(∆L*\right)}^{2}+{\left(∆a*\right)}^{2}+{\left(∆b*\right)}^{2}}$$

Microbial analyses were conducted in duplicate under sterile conditions to assess the growth of total bacteria in the packaged orange juice during cold storage. Thus, 1 mL of orange juice was aseptically collected and diluted in tubes containing 9 mL of 0.1% sterile peptone water (*w*/*v*: peptone/water). The tubes were vortexed for approximately 1 min at room temperature, and serial dilutions were prepared. Subsequently, 1 mL of each dilution was added to Petri dishes for total bacterial growth, followed by the addition of 15 mL of plate count agar (PCA) for pour plating. The plates were incubated at 37 °C for 48 h, and the results were expressed as CFU/mL.

#### 3.6.2. Sunflower Oil Oxidation Analysis

The peroxide value (PV) and the formation of dienes and trienes were measured to evaluate the antioxidant properties of the films with and without extract (PLA and PLA-E160). For this purpose, 10 mL of sunflower oil was packaged in heat-sealed single-dose bags and stored for 0, 15, 30, and 45 days under accelerated conditions (40 °C, 53% RH, and fluorescent light at 1000–1500 lux) (Luxometer model RS ILM1332A, RS Components). As a control, an open glass Petri dish containing 10 mL of sunflower oil was used.

To determine the PV of the samples, a titrimetric method using an automatic titrator (Titrando, Metrohm Ion Analysis, Herisau, Switzerland) was employed. Briefly, 1 g of oil was weighed and dissolved in 10 mL of glacial acetic acid/1-decanol in a 3:2 (*v*/*v*) volume ratio and mixed with 200 μL of a supersaturated KI solution. The mixture was shaken and kept in the dark for 1 min. Then, 50 mL of distilled, boiled, and cooled water was added, and the solution was titrated with sodium thiosulfate pentahydrate (Na_2_S_2_O_3_·5H_2_O) of variable normality (0.1 N, 0.01 N, or 0.002 N), depending on the expected PV [55]. All analyses were performed in duplicate. 

Conjugated dienes and trienes were determined in triplicate using a spectrophotometric method according to European Regulation EC2568/91. Absorbance was measured in adequately diluted samples in isooctane using a spectrophotometer (Evolution 201 UV-Visible Spectrophotometer, ThermoScientific, Dreieich, Germany) at 232 nm (dienes) and 268 nm (trienes). To calculate the concentration of dienes and trienes, the following equation was used:

(4) $$K_{\lambda }=\frac{E_{\lambda }}{c\times s}$$

where *K_λ_* is the specific extinction coefficient at the determined wavelength (λ), *E_λ_* is the absorbance at the determined wavelength, *c* is the concentration of the solution in g/100 mL, and *s* is the thickness of the quartz cuvettes in cm.

### 3.7. Statistical Analysis

Statistical analysis was performed using a one-way analysis of variance (ANOVA) with Statgraphics Centurión XVIII (StatgraphicsTechnologies, Inc., Rockville, MD, USA). Fisher’s least significant difference (LSD) test was used at a 95% confidence level to determine significant differences.

## 4. Conclusions

The incorporation of the peel almond (PA) extracts led to significant changes (*p* < 0.05) in the appearance, microstructure, and physical properties of PLA films. The extract-rich films were mechanically weaker, exhibited lower thermal stability, and had a darker brownish appearance compared to the neat PLA films. These changes can be partially attributed to their heterogeneous microstructure due to the presence of dispersed aggregates and small cavities. In contrast, the incorporation of the PA extracts did not affect the film’s barrier properties. The formation of new compounds, such as HFM, was detected during both the extraction process and the subsequent thermo-processing of the films. The release of phenolic compounds and HFM was significantly affected by the food simulant used, being promoted in the more polar simulant due to matrix swelling and relaxation and being reduced when using water or acidic simulants. Regarding the potential migration of HMF into real foodstuffs, it is concluded that PLA films containing active extracts can be considered safe.

The incorporation of almond peel extract into PLA films played a significant role in preserving the quality of both orange juice and sunflower oil, primarily by limiting oxidative reactions. The higher effectiveness of PLA-160 active films in preventing the oxidation of sunflower oil, compared to orange juice, is consistent with the limited release of active compounds in acidic, high-polarity simulants. These results highlight the potential antioxidant activity of PLA films incorporating almond peel extracts and may therefore be of interest for other food products sensitive to oxidative reactions.

## Figures and Tables

**Table 1:** Total phenolic (TPC) and ash contents, antioxidant activity (EC_50_), and minimal inhibitory concentration (MIC) of the extracts of defatted almond peels obtained by SWE at 160 and 180 °C (E-160 and E-180) (values ± standard deviation). Adapted from [6].

	E-160	E-180
TPC_1_ (g GAE. 100 g^−1^ dried extract)	10.09 ± 0.05 ^b^	16.1 ± 0.4 ^a^
Protein (g 100 g^−1^ dried extract)	2.4 ± 0.1 ^a^	2.2 ± 0.1 ^a^
Ashes (g 100 g^−1^ dried extract)	13.1 ± 0.1 ^a^	15.5 ± 0.7 ^b^
Carbohydrates (g 100 g^−1^ dried extract) *	74.4 ±0.2 ^a^	66.2 ± 0.2 ^b^
EC_50_ (mg extract.mg^−1^ DPPH)	1.490 ± 0.003 ^a^	1.063 ± 0.012 ^b^
MIC *L. innocua* (mg.mL^−1^)	90	34
MIC *E. coli* (mg.mL^−1^)	90	48
MIC *S. aureus* (mg.mL^−1^)	60	20

^a,b^ Different letters in the same row indicate significant differences between films (α = 0.05). * calculated by difference.

**Table 2:** Thickness values, moisture content, mechanical strength (TS), elasticity modulus (EM), elongation at break (E), water vapor, and oxygen permeabilities of the different films. Mean values and standard deviation.

Property	PLA	PLA-E160	PLA-E180
Thickness (μm)	134 ± 15 ^ab^	141 ± 12 ^a^	131 ± 8 ^b^
Xw (%)	2.2 ± 0.8 ^a^	1.07 ± 0.03 ^a^	1.4 ± 0.4 ^a^
EM (MPa)	1564 ± 42 ^a^	1461 ± 70 ^a^	1244 ± 102 ^b^
TS (MPa)	48 ± 2 ^a^	41 ± 2 ^b^	8 ± 4 ^c^
E%	3.2 ± 0.2 ^a^	2.9 ± 0.1 ^b^	0.5 ± 0.2 ^c^
WVP × 10^11^ (g/Pa.s.m)	2.6 ± 0.6 ^a^	3.6 ± 0.2 ^a^	2.3 ± 0.1 ^a^
OP × 10^14^ (cm^3^/m.s.Pa)	124 ± 1 ^a^	121 ± 6 ^a^	111 ± 4 ^b^
T onset (°C)	323.5 ± 1.1 ^a^	297 ± 4 ^b^	263 ± 5 ^c^
T peak (°C)	354.2 ± 0.5 ^a^	342.17 ± 0.01 ^b^	327.8 ± 2 ^c^
Tg_1_ (°C)	58.8 ± 0.5 ^a^	56.2 ± 0.2 ^a^	53 ± 3 ^a^
Tg_2_ (°C)	55.06 ± 0.1 ^a^	53.2 ± 0.6 ^b^	52.3 ± 0.5 ^b^
Log (UFC/mL) *L. innocua*	7.62	7.57	7.54
Log (UFC/mL) *E. coli*	8.10	8.08	7.97
Log (UFC/mL) *S. aureus*	8.04	7.82	7.81

^a,b,c^ Different letters in the same row indicate significant differences between films (α = 0.05).

**Table 3:** Hydroximethylfurfural migrated from the films to the different food simulants at 25 °C, expressed as mg/Kg, by considering a cubic package of 1 dm side.

HFM Released (ppm)	Water	Acetic Acid	10% Ethanol	95% Ethanol
PLA-E160	0.032 ± 0.008 ^d,2^	0.11 ± 0.01 ^b,2^	0.094 ± 0.009 ^b,2^	0.35 ± 0.04 ^a,2^
PLA-E180	0.0147 ± 0.001 ^d,1^	0.154 ± 0.009 ^c,1^	0.21 ± 0.03 ^c,1^	0.474 ± 0.002 ^a,1^

^a,b,c,d^ Different superscript indicates significant differences among simulants for the same film (*p* < 0.05). ^1,2^ Different superscript indicates significant differences among films for the same simulant (*p* < 0.05).

## Data Availability

Data is contained within the article.

## Supplementary Materials

The following supporting information can be downloaded at: https://www.mdpi.com/article/10.3390/molecules30091988/s1, Table S1: Lightness (L*), hue (h*ab) and chroma (C*ab) values of the orange juice packaged in the PLA and PLA-E160 films throughout cold storage, along with the total colour difference with respect to the fresh juice. (mean and standard deviation).
