# Supplementary material for: Active Polylactic Acid (PLA) Films Incorporating Almond Peel Extracts for Food Preservation
Source: Molecules. 2025 Apr 29;30(9):1988. doi: 10.3390/molecules30091988 (PMC12073344; doi:10.3390/molecules30091988)
Supplement: Supplementary file 1 [file molecules-30-01988-s001.zip › molecules-3553172-supplementary.pdf]

Table S1. Lightness (L*), hue (h*ab) and chroma (C*ab) values of the orange juice packaged in the PLA and PLA-E160 films throughout cold storage, along with the total colour difference with respect to the fresh juice. (mean and standard deviation)

Sample	Time (days)	L*	h*ab	C*ab	∆E
Fresh juice	0	63,35±0,2^{1a}	80,05±0,01^{1a}	70,62±0,03^{1a}	-
PLA	3	62,18±0,04^{2a}	81,36±0,01^{2a}	70,24±0,02^{2a}	2,04±0,01^{1,a}
PLA	7	59,52±0,1^{3a}	81,77±0,02^{3a}	71,02±0,16^{3a}	4,4±0,1^{2,a}
PLA	14	58,24±0,04^{4a}	81,68±0,01^{4a}	71,64±0,14^{4a}	5,60±0,05^{3,a}
PLA-E160	3	61,85±0,07^{2b}	80,88±0,02^{2b}	71,05±0,06^{2b}	1,87±0,06^{1,a}
PLA-E160	7	60,47±0,04^{3b}	81,04±0,01^{3b}	69,42±0,01^{3b}	3,36±0,04^{2,b}
PLA-E160	14	58,19±0,03^{4a}	81,06±0,01^{3b}	71,4±0,3^{4a}	5,37±0,02^{3,b}

1,2,3..different numbers in the same column indicate significant differences among storage time for a given sample ($p<0.05$)
a,b,c…different letters in the same column indicate significant differences among samples fro a given storage time ($p<0.05$).
